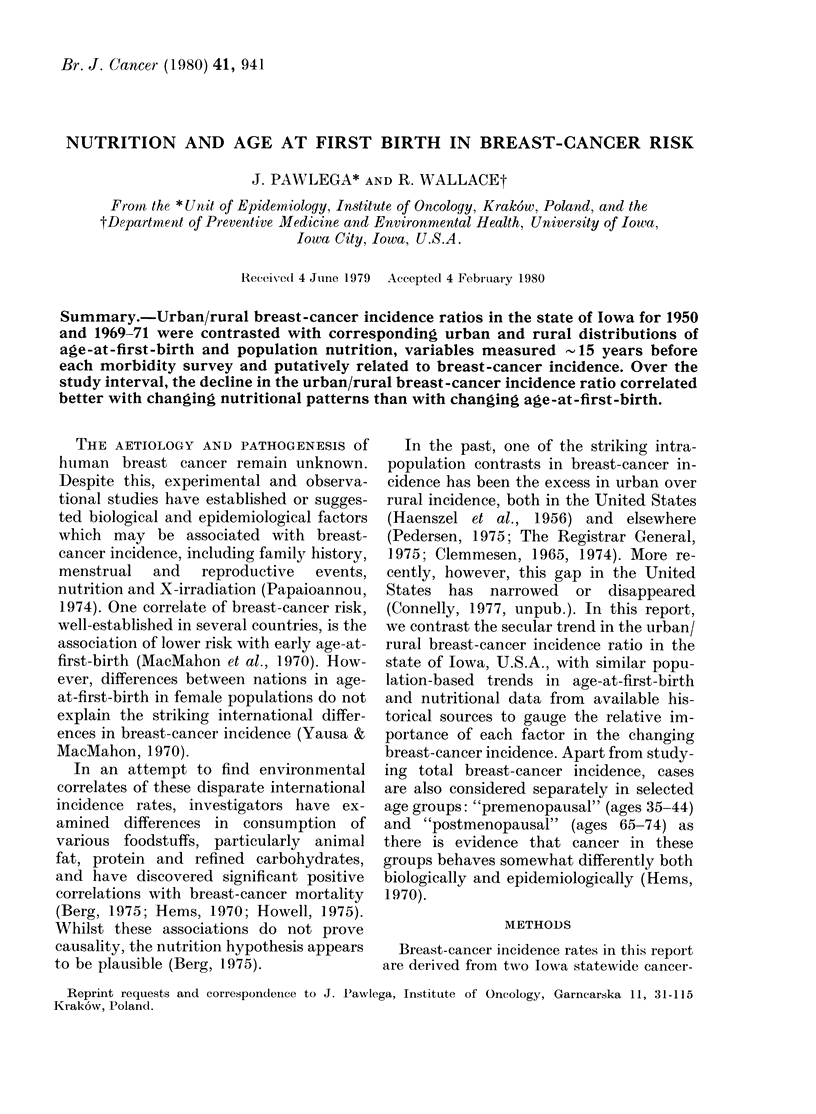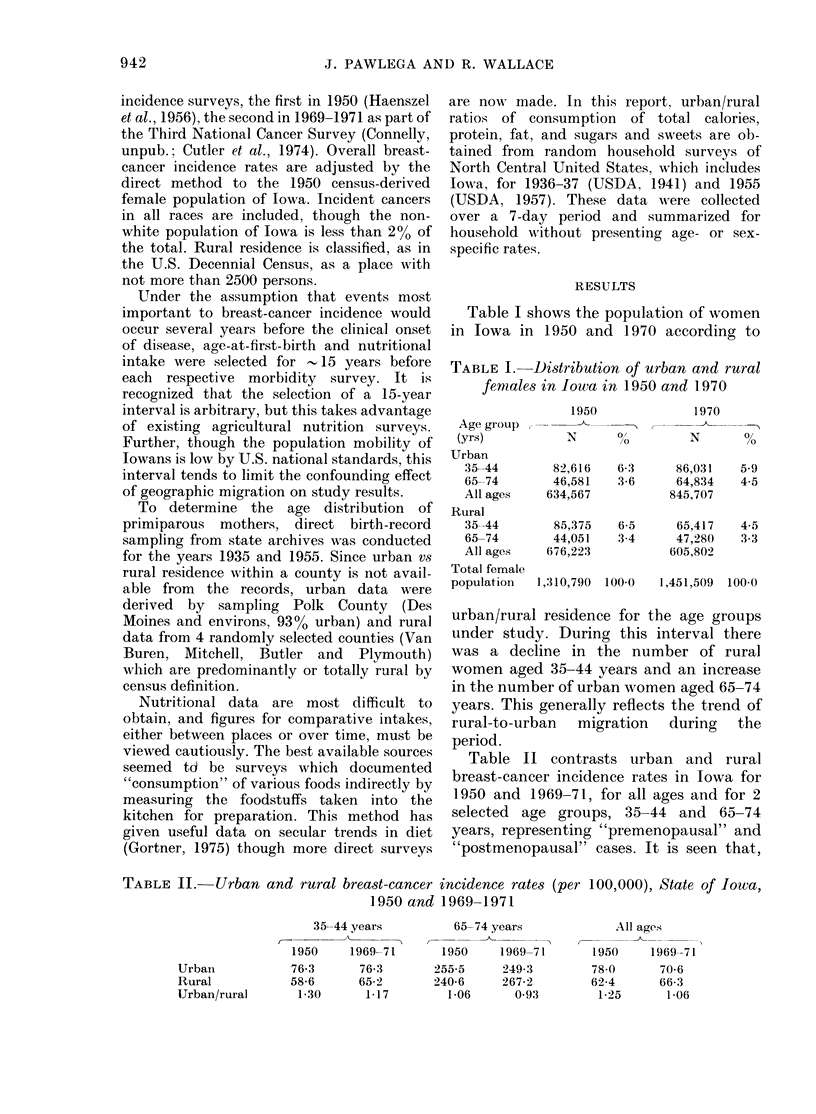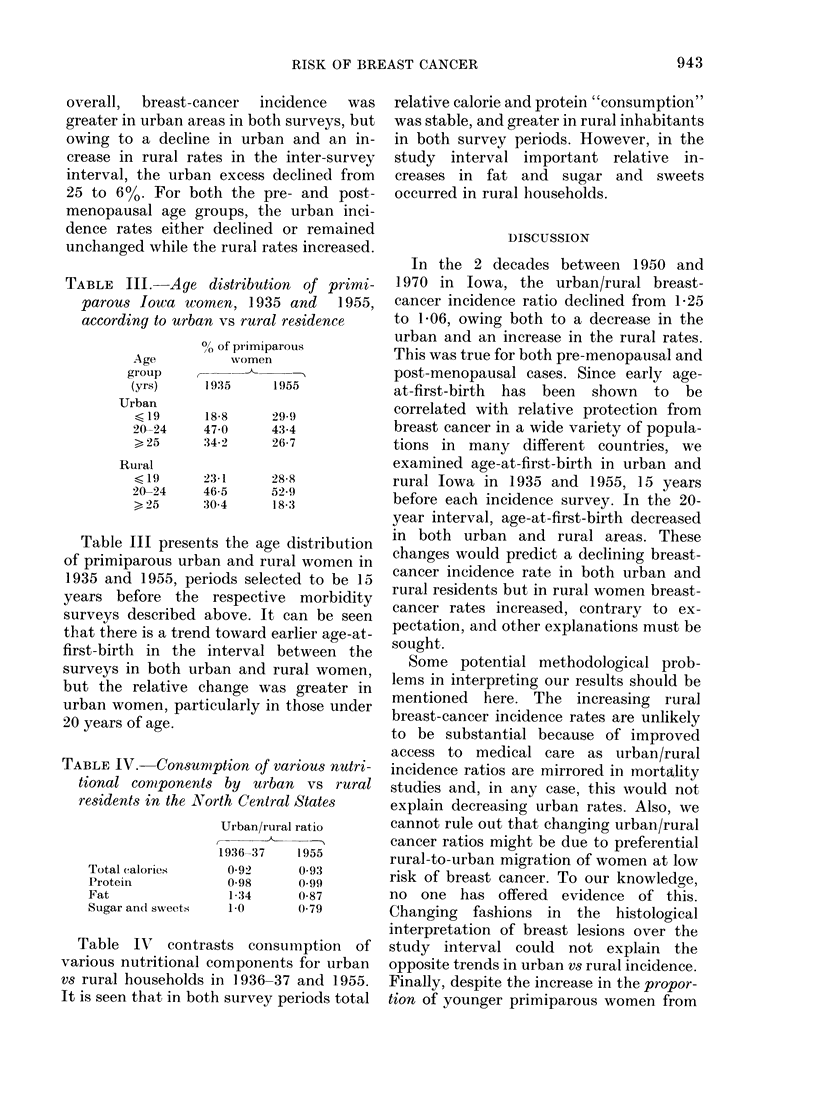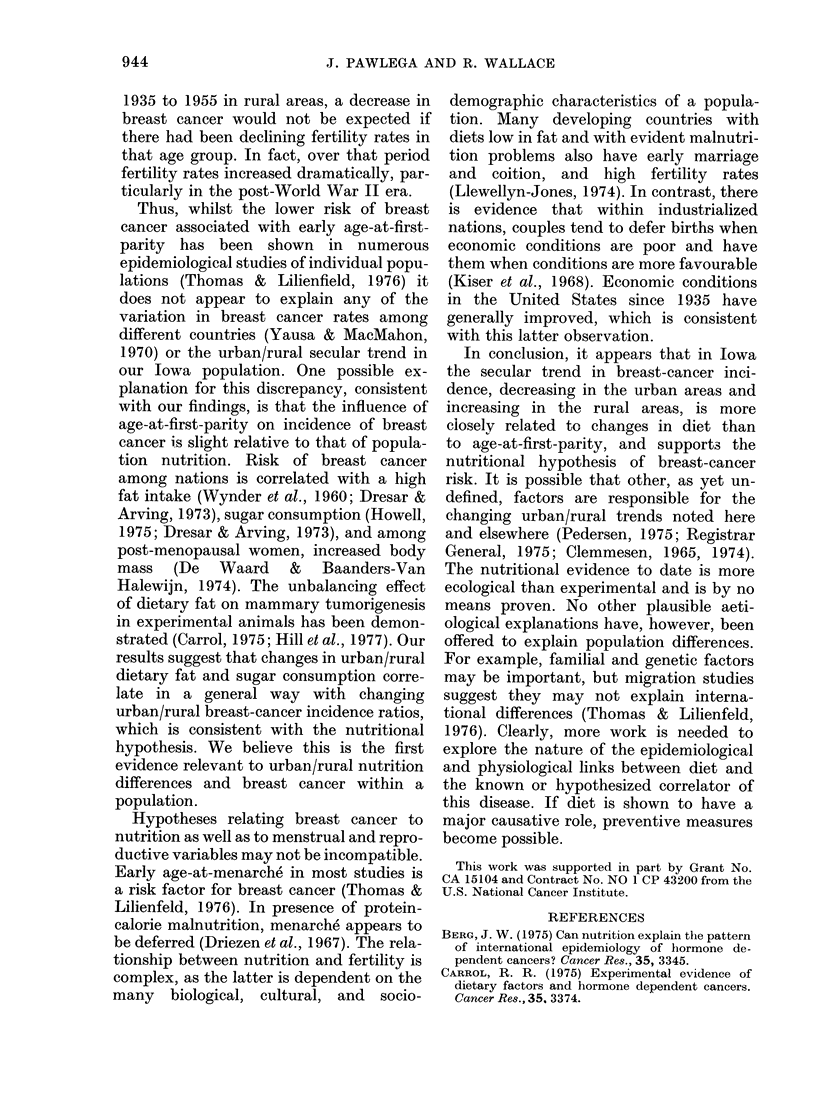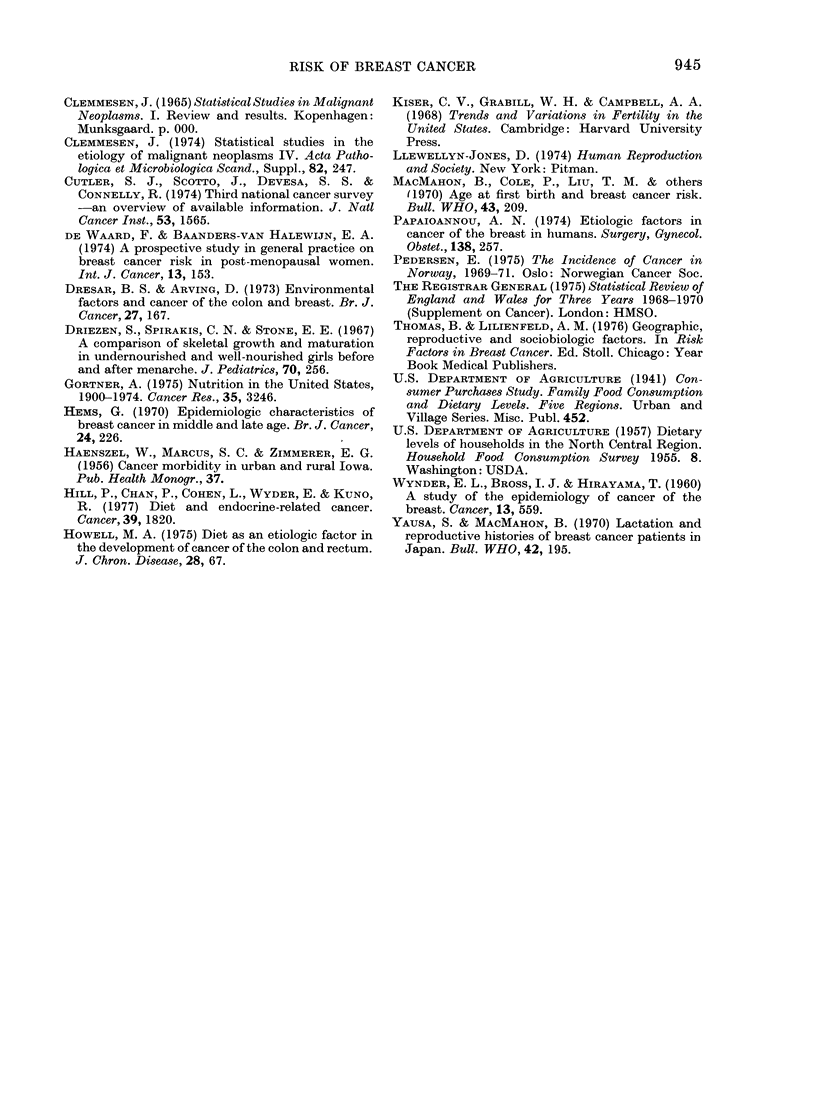# Nutrition and age at first birth in breast-cancer risk.

**DOI:** 10.1038/bjc.1980.172

**Published:** 1980-06

**Authors:** J. Pawlega, R. Wallace

## Abstract

Urban/rural breast-cancer incidence ratios in the state of Iowa for 1950 and 1969-71 were contrasted with corresponding urban and rural distributions of age-at-first-birth and population nutrition, variable measured approximately 15 years before each morbidity survey and putatively related to breast-cancer incidence. Over the study interval, the decline in the urban/rural breast-cancer incidence ratio correlated better with changing nutritional patterns than with changing age-at-first-birth.


*Br. J. Cancer* (1980) **41**, 941

### NUTRITION AND AGE AT FIRST BIRTH IN BREAST-CANCER RISK

J. PAWLEGA* AND R. WALLACE†

*From the *Unit of Epidemiology, Institute of Oncology, Kraków, Poland, and the †Department of Preventive Medicine and Environmental Health, University of Iowa, Iowa City, Iowa, U.S.A.*

Received 4 June 1979 Accepted 4 February 1980

**Summary.—Urban/rural breast-cancer incidence ratios in the state of Iowa for 1950 and 1969–71 were contrasted with corresponding urban and rural distributions of age-at-first-birth and population nutrition, variables measured ~15 years before each morbidity survey and putatively related to breast-cancer incidence. Over the study interval, the decline in the urban/rural breast-cancer incidence ratio correlated better with changing nutritional patterns than with changing age-at-first-birth.**

THE AETIOLOGY AND PATHOGENESIS of human breast cancer remain unknown. Despite this, experimental and observational studies have established or suggested biological and epidemiological factors which may be associated with breast-cancer incidence, including family history, menstrual and reproductive events, nutrition and X-irradiation (Papaioannou, 1974). One correlate of breast-cancer risk, well-established in several countries, is the association of lower risk with early age-at-first-birth (MacMahon *et al.*, 1970). However, differences between nations in age-at-first-birth in female populations do not explain the striking international differences in breast-cancer incidence (Yausa & MacMahon, 1970).

In an attempt to find environmental correlates of these disparate international incidence rates, investigators have examined differences in consumption of various foodstuffs, particularly animal fat, protein and refined carbohydrates, and have discovered significant positive correlations with breast-cancer mortality (Berg, 1975; Hems, 1970; Howell, 1975). Whilst these associations do not prove causality, the nutrition hypothesis appears to be plausible (Berg, 1975).

In the past, one of the striking intra-population contrasts in breast-cancer incidence has been the excess in urban over rural incidence, both in the United States (Haenszel *et al.*, 1956) and elsewhere (Pedersen, 1975; The Registrar General, 1975; Clemmesen, 1965, 1974). More recently, however, this gap in the United States has narrowed or disappeared (Connelly, 1977, unpub.). In this report, we contrast the secular trend in the urban/rural breast-cancer incidence ratio in the state of Iowa, U.S.A., with similar population-based trends in age-at-first-birth and nutritional data from available historical sources to gauge the relative importance of each factor in the changing breast-cancer incidence. Apart from studying total breast-cancer incidence, cases are also considered separately in selected age groups: "premenopausal" (ages 35–44) and "postmenopausal" (ages 65–74) as there is evidence that cancer in these groups behaves somewhat differently both biologically and epidemiologically (Hems, 1970).

### METHODS

Breast-cancer incidence rates in this report are derived from two Iowa statewide cancer-

Reprint requests and correspondence to J. Pawlega, Institute of Oncology, Garncarska 11, 31-115 Kraków, Poland.

incidence surveys, the first in 1950 (Haenszel *et al.*, 1956), the second in 1969–1971 as part of the Third National Cancer Survey (Connelly, unpub.; Cutler *et al.*, 1974). Overall breast-cancer incidence rates are adjusted by the direct method to the 1950 census-derived female population of Iowa. Incident cancers in all races are included, though the non-white population of Iowa is less than 2% of the total. Rural residence is classified, as in the U.S. Decennial Census, as a place with not more than 2500 persons.

Under the assumption that events most important to breast-cancer incidence would occur several years before the clinical onset of disease, age-at-first-birth and nutritional intake were selected for ~15 years before each respective morbidity survey. It is recognized that the selection of a 15-year interval is arbitrary, but this takes advantage of existing agricultural nutrition surveys. Further, though the population mobility of Iowans is low by U.S. national standards, this interval tends to limit the confounding effect of geographic migration on study results.

To determine the age distribution of primiparous mothers, direct birth-record sampling from state archives was conducted for the years 1935 and 1955. Since urban *vs* rural residence within a county is not available from the records, urban data were derived by sampling Polk County (Des Moines and environs, 93% urban) and rural data from 4 randomly selected counties (Van Buren, Mitchell, Butler and Plymouth) which are predominantly or totally rural by census definition.

Nutritional data are most difficult to obtain, and figures for comparative intakes, either between places or over time, must be viewed cautiously. The best available sources seemed to be surveys which documented "consumption" of various foods indirectly by measuring the foodstuffs taken into the kitchen for preparation. This method has given useful data on secular trends in diet (Gortner, 1975) though more direct surveys are now made. In this report, urban/rural ratios of consumption of total calories, protein, fat, and sugars and sweets are obtained from random household surveys of North Central United States, which includes Iowa, for 1936–37 (USDA, 1941) and 1955 (USDA, 1957). These data were collected over a 7-day period and summarized for household without presenting age- or sex-specific rates.

### RESULTS

Table I shows the population of women in Iowa in 1950 and 1970 according to urban/rural residence for the age groups under study. During this interval there was a decline in the number of rural women aged 35–44 years and an increase in the number of urban women aged 65–74 years. This generally reflects the trend of rural-to-urban migration during the period.

TABLE I.—*Distribution of urban and rural females in Iowa in* 1950 *and* 1970

| Age group (yrs) | 1950 N | 1950 % | 1970 N | 1970 % |
|---|---|---|---|---|
| Urban | | | | |
| 35–44 | 82,616 | 6·3 | 86,031 | 5·9 |
| 65–74 | 46,581 | 3·6 | 64,834 | 4·5 |
| All ages | 634,567 | | 845,707 | |
| Rural | | | | |
| 35–44 | 85,375 | 6·5 | 65,417 | 4·5 |
| 65–74 | 44,051 | 3·4 | 47,280 | 3·3 |
| All ages | 676,223 | | 605,802 | |
| Total female population | 1,310,790 | 100·0 | 1,451,509 | 100·0 |

Table II contrasts urban and rural breast-cancer incidence rates in Iowa for 1950 and 1969–71, for all ages and for 2 selected age groups, 35–44 and 65–74 years, representing "premenopausal" and "postmenopausal" cases. It is seen that,

TABLE II.—*Urban and rural breast-cancer incidence rates* (*per* 100,000), *State of Iowa,* 1950 *and* 1969–1971

| | 35–44 years | | 65–74 years | | All ages | |
|---|---|---|---|---|---|---|
| | 1950 | 1969–71 | 1950 | 1969–71 | 1950 | 1969–71 |
| Urban | 76·3 | 76·3 | 255·5 | 249·3 | 78·0 | 70·6 |
| Rural | 58·6 | 65·2 | 240·6 | 267·2 | 62·4 | 66·3 |
| Urban/rural | 1·30 | 1·17 | 1·06 | 0·93 | 1·25 | 1·06 |

overall, breast-cancer incidence was greater in urban areas in both surveys, but owing to a decline in urban and an increase in rural rates in the inter-survey interval, the urban excess declined from 25 to 6%. For both the pre- and post-menopausal age groups, the urban incidence rates either declined or remained unchanged while the rural rates increased.

TABLE III.—*Age distribution of primiparous Iowa women,* 1935 *and* 1955, *according to urban* vs *rural residence*

| Age group (yrs) | % of primiparous women | |
|---|---|---|
| | 1935 | 1955 |
| Urban | | |
| ≤19 | 18·8 | 29·9 |
| 20–24 | 47·0 | 43·4 |
| ≥25 | 34·2 | 26·7 |
| Rural | | |
| ≤19 | 23·1 | 28·8 |
| 20–24 | 46·5 | 52·9 |
| ≥25 | 30·4 | 18·3 |

Table III presents the age distribution of primiparous urban and rural women in 1935 and 1955, periods selected to be 15 years before the respective morbidity surveys described above. It can be seen that there is a trend toward earlier age-at-first-birth in the interval between the surveys in both urban and rural women, but the relative change was greater in urban women, particularly in those under 20 years of age.

TABLE IV.—*Consumption of various nutritional components by urban* vs *rural residents in the North Central States*

| | Urban/rural ratio | |
|---|---|---|
| | 1936–37 | 1955 |
| Total calories | 0·92 | 0·93 |
| Protein | 0·98 | 0·99 |
| Fat | 1·34 | 0·87 |
| Sugar and sweets | 1·0 | 0·79 |

Table IV contrasts consumption of various nutritional components for urban *vs* rural households in 1936–37 and 1955. It is seen that in both survey periods total relative calorie and protein "consumption" was stable, and greater in rural inhabitants in both survey periods. However, in the study interval important relative increases in fat and sugar and sweets occurred in rural households.

### DISCUSSION

In the 2 decades between 1950 and 1970 in Iowa, the urban/rural breast-cancer incidence ratio declined from 1·25 to 1·06, owing both to a decrease in the urban and an increase in the rural rates. This was true for both pre-menopausal and post-menopausal cases. Since early age-at-first-birth has been shown to be correlated with relative protection from breast cancer in a wide variety of populations in many different countries, we examined age-at-first-birth in urban and rural Iowa in 1935 and 1955, 15 years before each incidence survey. In the 20-year interval, age-at-first-birth decreased in both urban and rural areas. These changes would predict a declining breast-cancer incidence rate in both urban and rural residents but in rural women breast-cancer rates increased, contrary to expectation, and other explanations must be sought.

Some potential methodological problems in interpreting our results should be mentioned here. The increasing rural breast-cancer incidence rates are unlikely to be substantial because of improved access to medical care as urban/rural incidence ratios are mirrored in mortality studies and, in any case, this would not explain decreasing urban rates. Also, we cannot rule out that changing urban/rural cancer ratios might be due to preferential rural-to-urban migration of women at low risk of breast cancer. To our knowledge, no one has offered evidence of this. Changing fashions in the histological interpretation of breast lesions over the study interval could not explain the opposite trends in urban *vs* rural incidence. Finally, despite the increase in the *proportion* of younger primiparous women from

1935 to 1955 in rural areas, a decrease in breast cancer would not be expected if there had been declining fertility rates in that age group. In fact, over that period fertility rates increased dramatically, particularly in the post-World War II era.

Thus, whilst the lower risk of breast cancer associated with early age-at-first-parity has been shown in numerous epidemiological studies of individual populations (Thomas & Lilienfield, 1976) it does not appear to explain any of the variation in breast cancer rates among different countries (Yausa & MacMahon, 1970) or the urban/rural secular trend in our Iowa population. One possible explanation for this discrepancy, consistent with our findings, is that the influence of age-at-first-parity on incidence of breast cancer is slight relative to that of population nutrition. Risk of breast cancer among nations is correlated with a high fat intake (Wynder *et al.*, 1960; Dresar & Arving, 1973), sugar consumption (Howell, 1975; Dresar & Arving, 1973), and among post-menopausal women, increased body mass (De Waard & Baanders-Van Halewijn, 1974). The unbalancing effect of dietary fat on mammary tumorigenesis in experimental animals has been demonstrated (Carrol, 1975; Hill *et al.*, 1977). Our results suggest that changes in urban/rural dietary fat and sugar consumption correlate in a general way with changing urban/rural breast-cancer incidence ratios, which is consistent with the nutritional hypothesis. We believe this is the first evidence relevant to urban/rural nutrition differences and breast cancer within a population.

Hypotheses relating breast cancer to nutrition as well as to menstrual and reproductive variables may not be incompatible. Early age-at-menarché in most studies is a risk factor for breast cancer (Thomas & Lilienfeld, 1976). In presence of protein-calorie malnutrition, menarché appears to be deferred (Driezen *et al.*, 1967). The relationship between nutrition and fertility is complex, as the latter is dependent on the many biological, cultural, and socio-demographic characteristics of a population. Many developing countries with diets low in fat and with evident malnutrition problems also have early marriage and coition, and high fertility rates (Llewellyn-Jones, 1974). In contrast, there is evidence that within industrialized nations, couples tend to defer births when economic conditions are poor and have them when conditions are more favourable (Kiser *et al.*, 1968). Economic conditions in the United States since 1935 have generally improved, which is consistent with this latter observation.

In conclusion, it appears that in Iowa the secular trend in breast-cancer incidence, decreasing in the urban areas and increasing in the rural areas, is more closely related to changes in diet than to age-at-first-parity, and supports the nutritional hypothesis of breast-cancer risk. It is possible that other, as yet undefined, factors are responsible for the changing urban/rural trends noted here and elsewhere (Pedersen, 1975; Registrar General, 1975; Clemmesen, 1965, 1974). The nutritional evidence to date is more ecological than experimental and is by no means proven. No other plausible aetiological explanations have, however, been offered to explain population differences. For example, familial and genetic factors may be important, but migration studies suggest they may not explain international differences (Thomas & Lilienfeld, 1976). Clearly, more work is needed to explore the nature of the epidemiological and physiological links between diet and the known or hypothesized correlator of this disease. If diet is shown to have a major causative role, preventive measures become possible.

This work was supported in part by Grant No. CA 15104 and Contract No. NO 1 CP 43200 from the U.S. National Cancer Institute.

### REFERENCES

BERG, J. W. (1975) Can nutrition explain the pattern of international epidemiology of hormone dependent cancers? *Cancer Res.*, **35**, 3345.

CARROL, R. R. (1975) Experimental evidence of dietary factors and hormone dependent cancers. *Cancer Res.*, **35**, 3374.

CLEMMESEN, J. (1965) *Statistical Studies in Malignant Neoplasms.* I. Review and results. Kopenhagen: Munksgaard. p. 000.

CLEMMESEN, J. (1974) Statistical studies in the etiology of malignant neoplasms IV. *Acta Pathologica et Microbiologica Scand.*, Suppl., **82**, 247.

CUTLER, S. J., SCOTTO, J., DEVESA, S. S. & CONNELLY, R. (1974) Third national cancer survey—an overview of available information. *J. Natl Cancer Inst.*, **53**, 1565.

DE WAARD, F. & BAANDERS-VAN HALEWIJN, E. A. (1974) A prospective study in general practice on breast cancer risk in post-menopausal women. *Int. J. Cancer*, **13**, 153.

DRESAR, B. S. & ARVING, D. (1973) Environmental factors and cancer of the colon and breast. *Br. J. Cancer*, **27**, 167.

DRIEZEN, S., SPIRAKIS, C. N. & STONE, E. E. (1967) A comparison of skeletal growth and maturation in undernourished and well-nourished girls before and after menarche. *J. Pediatrics*, **70**, 256.

GORTNER, A. (1975) Nutrition in the United States, 1900–1974. *Cancer Res.*, **35**, 3246.

HEMS, G. (1970) Epidemiologic characteristics of breast cancer in middle and late age. *Br. J. Cancer*, **24**, 226.

HAENSZEL, W., MARCUS, S. C. & ZIMMERER, E. G. (1956) Cancer morbidity in urban and rural Iowa. *Pub. Health Monogr.*, **37**.

HILL, P., CHAN, P., COHEN, L., WYDER, E. & KUNO, R. (1977) Diet and endocrine-related cancer. *Cancer*, **39**, 1820.

HOWELL, M. A. (1975) Diet as an etiologic factor in the development of cancer of the colon and rectum. *J. Chron. Disease*, **28**, 67.

KISER, C. V., GRABILL, W. H. & CAMPBELL, A. A. (1968) *Trends and Variations in Fertility in the United States.* Cambridge: Harvard University Press.

LLEWELLYN-JONES, D. (1974) *Human Reproduction and Society.* New York: Pitman.

MACMAHON, B., COLE, P., LIU, T. M. & others (1970) Age at first birth and breast cancer risk. *Bull. WHO*, **43**, 209.

PAPAIOANNOU, A. N. (1974) Etiologic factors in cancer of the breast in humans. *Surgery, Gynecol. Obstet.*, **138**, 257.

PEDERSEN, E. (1975) *The Incidence of Cancer in Norway*, 1969–71. Oslo: Norwegian Cancer Soc.

THE REGISTRAR GENERAL (1975) *Statistical Review of England and Wales for Three Years* 1968–1970 (Supplement on Cancer). London: HMSO.

THOMAS, B. & LILIENFELD, A. M. (1976) Geographic, reproductive and sociobiologic factors. In *Risk Factors in Breast Cancer.* Ed. Stoll. Chicago: Year Book Medical Publishers.

U.S. DEPARTMENT OF AGRICULTURE (1941) *Consumer Purchases Study. Family Food Consumption and Dietary Levels. Five Regions.* Urban and Village Series. Misc. Publ. **452**.

U.S. DEPARTMENT OF AGRICULTURE (1957) Dietary levels of households in the North Central Region. *Household Food Consumption Survey* 1955. **8**. Washington: USDA.

WYNDER, E. L., BROSS, I. J. & HIRAYAMA, T. (1960) A study of the epidemiology of cancer of the breast. *Cancer*, **13**, 559.

YAUSA, S. & MACMAHON, B. (1970) Lactation and reproductive histories of breast cancer patients in Japan. *Bull. WHO*, **42**, 195.